# Determination of the optimal dose of ephedrine in the treatment of arterial hypotension due to general anesthesia in neonates and infants below 6 months old: the ephedrine study protocol for a randomized, open-label, controlled, dose escalation trial

**DOI:** 10.1186/s13063-021-05155-2

**Published:** 2021-03-12

**Authors:** A. S. Szostek, P. Boucher, F. Subtil, O. Zerzaihi, C. Saunier, M. de Queiroz Siqueira, F. Merquiol, P. Martin, M. Granier, A. Gerst, A. Lambert, T. Storme, D. Chassard, P. Nony, B. Kassai, S. Gaillard

**Affiliations:** 1 Hospices Civils de Lyon, Service d’anesthésie pédiatrique-HFME, 69677 Bron, France; 2 Hospices Civils de Lyon, Service de Biostatistiques, Lyon, France; 3 Hospices Civils de Lyon, EPICIME-CIC 1407 de Lyon, Inserm, Département d’épidémiologie clinique, Bron, CHU-Lyon F-69677 France; 4 Department of Anesthesiology and Intensive Care, University Hospital of Saint-Etienne, Saint-Etienne Cedex, France; 5 Département de Médecine Périopératoire, Anesthésie et Réanimation, Centre Hospitalier Universitaire Clermont-Ferrand, 63000 Clermont-Ferrand, France; 6 Université de Lyon; CNRS, UMR 5558, Laboratoire de Biométrie et Biologie Evolutive, F-69622 Villeurbanne, France

**Keywords:** General anesthesia, Arterial hypotension, Optimal dose, Ephedrine, Randomized controlled trial

## Abstract

**Background:**

Arterial hypotension induced by general anesthesia is commonly identified as a risk factor of morbidity, especially neurological, after cardiac or noncardiac surgery in adults and children. Intraoperative hypotension is observed with sevoflurane anesthesia in children, in particular in neonates, infants younger than 6 months, and preterm babies. Ephedrine is commonly used to treat intraoperative hypotension. It is an attractive therapeutic, due to its dual action on receptors alpha and beta and its possible peripheral intravenous infusion. There are few data in the literature on the use of ephedrine in the context of pediatric anesthesia. The actual recommended dose of ephedrine (0.1 to 0.2 mg/Kg) frequently leads to a therapeutic failure in neonates and infants up to 6 months of age. The use of higher doses would probably lead to a better correction of hypotension in this population.

The objective of our project is to determine the optimal dose of ephedrine for the treatment of hypotension after induction of general anesthesia with sevoflurane, in neonates and infants up to 6 months of age.

**Methods:**

The ephedrine study is a prospective, randomized, open-label, controlled, dose-escalation trial. The dose escalation consists of 6 successive cohorts of 20 subjects. The doses studied are 0.6, 0.8, 1, 1.2, and 1.4 mg/kg. The dose chosen as the reference is 0.1 mg/kg, the actual recommended dose. Neonates and infants younger than 6 months, males and females, including preterm babies who undergo a surgery with general anesthesia inducted with sevoflurane were eligible. Parents of the subject were informed. Then, the subjects were randomized if presenting a decrease in mean blood pressure superior to 20% of their initial mean blood pressure (before induction of anesthesia), despite a vascular filling with sodium chloride 0.9%. The primary outcome is the success of the therapy defined as an mBP superior to 80% of the baseline mBP (prior to anesthesia) within 10 min post ephedrine administration. The subjects were followed-up for 3 days postanesthesia.

**Discussion:**

This study is the first randomized, controlled trial intending to determine the optimal dose of ephedrine to treat hypotension in neonates and infants below 6 months old.

**Trial registration:**

ClinicalTrials.gov NCT02384876. Registered on March 2015.

## Background

Practices in anesthesia have tremendously improved in pediatrics over the last decade but morbidity remains significant in the youngest patients below 1 year old. The two main complications are respiratory (desaturation, laryngospasm, bronchospasm) and hemodynamic (bradycardia, arterial hypotension, cardiorespiratory arrest). Sevoflurane is the most commonly used agent for the induction of general anesthesia (GA) in pediatric practice, because of its pharmacokinetic profile and its relative safety for the cardiopulmonary system [1]. Nevertheless, many episodes of idiopathic orthostatic hypotension (IOH) are routinely observed in children during GA with sevoflurane [2], in particular in neonates, infants younger than 6 months [3], and preterm babies [4]. Hypotension induced by GA is widely identified as a risk factor of morbidity, especially neurological, after cardiac or non-cardiac surgery in adults and children [5].

Avoiding and limiting this hypotension is a daily challenge for anesthesiologists. Intravascular volume expansion by crystalloid is the first recommended treatment when IOH occurs [6]. In case of failure, dopamine is widely recommended in infants younger than 6 months [7, 8]. Adrenaline administered continuously has similar effects but the access to central venous route is difficult. Thus, the use of adrenaline has then been restricted to severe conditions [3]. Ephedrine is an attractive drug to treat IOH, due to its dual action on receptors α and β and its use through peripheral intravenous route. Nevertheless, there are few data in the literature on the use of ephedrine in the context of pediatric anesthesia. Taguchi et al. have conducted a study from birth to adulthood. They showed that ephedrine (0.1 to 0.2 mg/kg) has a lower hemodynamic response in infants than in adults. Higher doses of ephedrine are probably needed because of the immaturity of the myocardium and sympathetic system, as already identified with dobutamine or dopamine [6, 9]. A recent retrospective cohort suggests an under efficacy of low doses and the need of higher doses than those recommended. This cohort gathered data on 141 subjects, aged 0 to 6.4 months (median = 1.41 months), having received a dose of ephedrine during GA under sevoflurane. Doses of ephedrine ranged between 0.07 and 1.33 mg/Kg (median = 0.25 mg/Kg). There was a great variability in the mean blood pressure (mBP) decrease after induction of anesthesia and in the response according to the dose of ephedrine. A non-linear mixed effects model was applied to estimate parameters of the sigmoid dose effect relationship, taking into account the variation of mBP and to estimate the effect of ephedrine according to the pre-induction mBP. After several simulations of clinical trials, the model suggested the use of higher doses, up to 2 mg/kg to reach a satisfactory efficacy (data not published). The model predicted that a dose of 1.4 mg/kg would allow 63% of patients with an mBP post ephedrine > 80% of the baseline mBP. Thus, a range of doses (0.2 to 1.4 mg/kg) was chosen to reach an objective of 55% of patients responding to the efficacy criteria compared to the reference dose of 0.1 mg/kg.

The usual recommended dose of ephedrine (0.1 to 0.2 mg/Kg) frequently leads to a therapeutic failure to treat hypotension in neonates and infants up to 6 months of age. The use of higher doses would probably lead to a better correction of hypotension in this population.

## Methods

### Objectives

The primary objective of our project is to determine the optimal dose of ephedrine to administer as a single dose, for the treatment of hypotension after induction of GA with sevoflurane, in neonates and infants up to 6 months of age.

As secondary objectives, we are describing the number of cases returning to a mean Blood pressure superior to 38 mmHg post ephedrine administration. We are also assessing the occurrence of hypoxemic events and the tolerance of ephedrine.

### Trial design

The ephedrine study is a prospective, randomized, open-label, controlled, dose-escalation trial *intending to demonstrate the superiority of a higher dose of ephedrine compared to the usual dose (0.1 to 0.2 mg/kg)*.

The dose escalation consists of 6 successive cohorts of 20 subjects. The doses studied are 0.6, 0.8, 1, 1.2, and 1.4 mg/kg. The dose chosen as the reference is 0.1 mg/kg, the usual recommended dose. After each completion of cohort, the tolerance profile is evaluated by an independent data monitoring committee. Recommendations are expressed for the continuation, modifications, or end of the study. To be able to take into account the cohort effect, an extended halving dose escalation design was used [10] as summarized in Table 1.

**Table 1:** Distribution of patients per cohort

**Dose (mg/kg)**	**0.1**	**0.6**	**0.8**	**1.0**	**1.2**	**1.4**
Cohort 1	10	10	0	0	0	0
Cohort 2	5	5	10	0	0	0
Cohort 3	3	3	4	10	0	0
Cohort 4	2	2	3	3	10	0
Cohort 5	2	2	2	2	2	10
Cohort 6	2	2	2	2	5	7
Total	24	24	21	17	17	17

### Study setting

This is a multicenter study conducted in three French university hospitals of the Auvergne-Rhône-Alpes area: Hôpital Femme Mère enfant - Hospices Civils de Lyon (HCL), Hôpital Estaing -CHU de Clermont-Ferrand, and Hôpital Nord – CHU de St-Etienne.

### Eligibility criteria

Neonates and infants younger than 6 months, males and females, including preterm babies, who undergo a surgery requiring GA inducted by inhaled sevoflurane and for whom parents/legal guardians have signed an informed consent are eligible. Subjects are not enrolled if presenting a known hypersensitivity to ephedrine, requiring a complex surgery, having received vasopressive amines different from ephedrine or other indirect sympathomimetic drugs (phenylpropanolamine, phenylephrine, pseudoephedrine and methylphenidate), pretreated with clonidine, suffering from a congenital cardiopathy, and not affiliated to a health insurance system.

Neonates and infants were randomized if presenting a decrease in mBP superior to 20% of their initial mBP (before induction of anesthesia), despite a vascular filling with saline solution 0.9% (10 ml/kg over 10 min).

### Intervention and allocation of study treatment

Study treatment is provided as 10 ml vials of ephedrine Aguettant®, 3 mg/mL. Packaging is per patient with a unique treatment number. Vials are distributed by a central pharmacy and available in the operating rooms of each site. The study intervention is a single intravenous administration of ephedrine. The tested doses are as follows: 0.6, 0.8, 1, 1.2, and 1.4 mg/kg. The reference treatment (control group) is ephedrine at a dose of 0.1 mg/kg (recommended dose, following summary of the product characteristics). The dose is prepared and administered by a different anesthesiologist than the one conducting GA. The 10 mL vial of ephedrine is diluted in 50 mL sodium chloride 0.9% to obtain a concentration of 0.5 mg/mL.

Subjects are randomized if a decrease in mBP superior to 20% of their baseline mBP is observed despite vascular filling with saline solution 0.9%. The randomization is centralized via ennov (Euraxipharma, CS online software). The dose and exact dilution to be prepared is indicated to the anesthesiologist by the software. Ephedrine is administered and a 10-min monitoring starts before surgery processes.

In case of treatment failure, the anesthesiologist can use another dose of ephedrine or any alternatives such as dopamine and adrenaline.

Only saline solution is authorized as vascular filling. GA induction with other volatile or IV anesthetic agents and treatment with hemodynamic effect such as clonidine and indirect sympathomimetic are forbidden.

### Outcomes

The primary endpoint is the change of mBP after administration of ephedrine. The therapeutic success is defined as an mBP superior to 80% of the initial mBP (prior induction of anesthesia) within 10 min post ephedrine administration. Another infusion of ephedrine or the administration of dopamine or adrenaline or a vascular filling within 10 min post initial dose of ephedrine is considered as a failure. Each case of surgery incisions happening before 10 min will be defined as success or failure at the blind review before analysis. The initial mBP is the average measure of 2 mBP recorded before anesthetic induction. If the difference between the two measurements is higher than 5%, a third measurement is performed. The mean of the 2 closest values defines the baseline mBP. BP is regularly monitored and recorded each minute for 10 min post-dose of ephedrine.

The secondary endpoints are as follows: (i) the continuous monitoring of BP to observe the number of patients returning to a mean blood pressure superior to 38 mmHg for 10 min post ephedrine administration; (ii) the evaluation of the changes in cerebral oxygen saturation (ScO^2^) measured by Near-Infrared Spectroscopy (NIRS, Oxyalert®, Covidien, US); hypoxic events (defined as decreases of ScO^2^ at an equal or lower level than the ScO^2^ level while subject is awake) are searched; frequency of desaturation and evolution of ScO^2^ under treatment are collected; and (iii) assessment of tolerance by monitoring of adverse events for the entire study participation, up to 3 days post-surgery. Adverse events expected with ephedrine such as cardiac events (tachycardia and arterial hypertension) and angle closure glaucoma are closely monitored.

### Participant timeline

The initial planned study duration is 24 months, 2 months of recruitment per cohort (i.e., 12 months), and 2 months of data analysis and review between each cohort (i.e., 12 months). The study duration per subject is 3 days post-randomization and the treatment is a single administration.

**Fig. 1:**
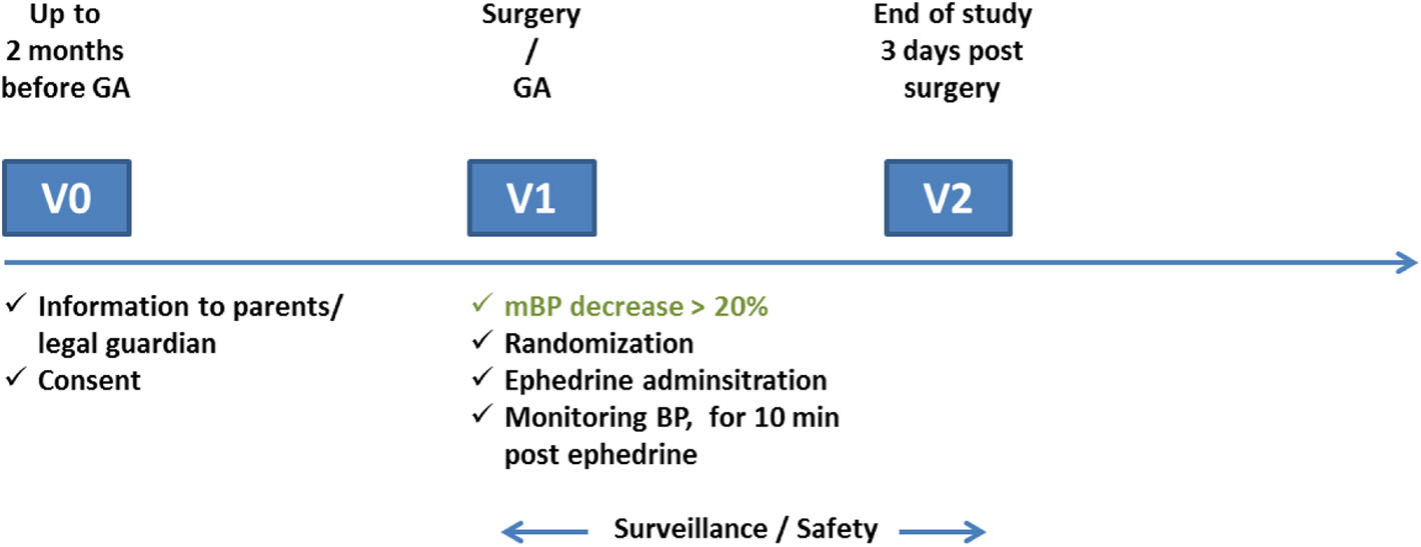
Ephedrine study visits

Figure 1 summarizes the study visits. Table 2 presents the intervention and evaluations performed.

**Table 2:** Ephedrine study interventions and assessments

	Study period	
	Enrolment	Allocation
***Timepoint***	***Pre-anesthesia visit***	General anesthesia
**Enrolment:**		
**Eligibility screen**	X	
**Informed consent**	X	X (check)
***General anesthesia***	X	X
**Randomization eligibility: decrease in mean blood pressure**		X
**Randomization**		X
**Interventions:**		
***Ephedrine 0.6, 0.8, 1.0, 1.2, 1.4%, tested doses***		X
***Ephedrine 0.1%, control group***		X
**Assessments:**		
***Systolic and diastolic blood pressure (baseline and every minute)***		X
***Mean blood pressure (baseline and every minute)***		X
***Heart rate, ScO2, PaCO2 every 10 min post ephedrine***		X
***Adverse events and concomitant medications***		X

### Data management

Outcomes related to hemodynamic measurements are collected within 10 min post ephedrine injection, as per site practice using a standard of care software. These data are usually collected during anesthesia. The investigators are checking that BP measurements are scheduled every 1 min. NIRS data are collected at the operative room, by the investigators, using paper documents.

Surveillance and emergence of adverse and serious adverse events is performed. A particular attention is given to expected adverse reactions.

All data collected in electronic or paper source documents are entered in an electronic case report form by a local study nurse. Data are coded with respect to data confidentiality. The Euraxipharma system was selected. Control quality of the data is performed both centrally and on site by monitors. Presence, accuracy, and conformity of the data are verified.

### Sample size

A total of 120 patients will be randomized, in 6 different cohorts with a maximal increasing dose.

The distribution of subjects as proposed in Table 1 for each dose and cohort should allow to maximizing the precision of the estimation of the differences of efficacy of each dose, taking into account the cohort effect, which could be significant in this study. This proposal is conform to the description of Bailey [10].

The sample size was calculated in terms of 95% confidence interval width of the probabilities of efficacy of each dose. The number of 120 subjects should allow width between 0.18 and 0.29, depending on the dose. These calculations are based on simulations performed using the R software; the hypotheses regarding the efficacy depending on the dose were based on results of the retrospective cohort on 141 subjects, considering a standard deviation of the cohort effect of 0.3 on a logarithmic scale of the odds of success.

### Statistical methods

All analyses will be performed on the per-protocol population, defined by all randomized subjects, having presented a decrease in their mBP superior to 20% of their baseline mBP and their actual dose of ephedrine received.

Qualitative data will be described by their numbers and percentages. Quantitative data will be described by their means, medians, standard deviations, minimum and maximum values, and first and third quartiles. Baseline characteristics of the patients will be presented per dose.

The analysis of the primary endpoint will use a mixed logistic regression model including a factor per dose and the random cohort effect. Variables of adjustment could be introduced in the model, as the change in arterial blood pressure under anesthesia from initial blood pressure. The odds ratio of efficacy of the doses compared to the reference dose will be estimated with their 95% confidence interval. The optimal dose will be the one with the odds ratio closest to the odds ratio equivalent to a difference of efficacy of 55% and for which the confidence interval is conform to this value. Exploratory secondary analyses of the primary endpoint could be performed by considering the dose as having a continuous effect.

The analysis of the return of the blood pressure above 38 mmHg, depending on the dose, will be performed similarly. Changes in cardiac frequency and ScO_2_, adverse events, and serious adverse events will be described per dose.

Missing data will not be replaced.

At completion of each cohort, analysis of tolerance will be performed. There will not be any statistical test.

## Discussion

This study is the first prospective randomized clinical trial intending to determine the optimal dose of ephedrine in the treatment of hypotension induced by anesthesia with sevoflurane, in neonates and infants from 0 to 6 months. Despite a great proportion of patients with IOH, the recruitment might be slower than expected, as parents/legal guardians are often refusing randomized clinical trials for their child [11]. The randomization at the operative room is also a challenge that is resolved with a dedicated web system.

In adult literature, mBP is a poor marker of cardiac output in perioperative period. However, for infants under 6 months, no monitoring devices are available and validated. So, we chose mBP as our primary endpoint and an mBP decrease higher than 20% for inclusion, in accordance with previous studies [2, 12].

This study has certain limitations. Our study population below 6 months old is not homogeneous. Evidence has shown that neonates below 2 months seem to have different maturity of the sympathetic system and myocardium, which could explain any difference in ephedrine efficiency according to the age of the child. This is why we chose to define therapeutic success/failure by a variation of mBP, and not with the absolute value of mBP.

mBP is monitored noninvasively in our study. This is not the gold standard, but this reflects common day-to-day practice. Moreover, very few children below 6 months of age are premedicated before surgery. It may increase baseline mBP values before GA. Children are exposed to the stress of a first blood pressure measurement while they are awake, which by default pumps the cuff to a pressure far above the real systolic BP, which can be a very unpleasant experience. Initial agitation of the child who will undergo surgery could increase by 2 or 3 the BP measurements and might overestimate the baseline BP. We may legitimately ask whether the drop of BP (and therefore therapeutic success) is not skewed by inaccurate reference values. Nevertheless, the non-invasively monitoring of BP is the reflection of day to day practice.

## Trial status

The current version of the protocol is version 5, May 7, 2019, amendment 3, authorized by the ethic committee on May 21, 2019. The competent authority was informed. First patient was enrolled on June 15, 2015. The last cohort started on December 6, 2019. One hundred and twenty subjects have been randomized in the study. Recruitment was completed in September 2020. The study was temporarily stopped during the COVID-19 pandemic, from March to May 2020. The trial protocol was not submitted at the beginning of the trial before inclusions, but registration in ClinicalTrials.gov was available at that time. The ephedrine protocol was submitted with a delay but with the wish to make available a complete protocol before statistical analysis of the study to allow checking the respect of the protocol. The protocol was submitted before end of study trial enrollment and before last patient last visit.

## Data Availability

At the end of the study, data will be available in the study database which is not publicly available. They cannot be provided until the end of the study but will then be available from the corresponding author, after approval of the sponsor, after thorough review of the scientific interest of the request by the study team.

## Abbreviations

BP: Blood pressure
GA: General anesthesia
IOH: Idiopathic orthostatic hypotension
mBP: Mean blood pressure
